# Treatment goals and changes over time in older patients with non-curable cancer

**DOI:** 10.1007/s00520-020-05945-5

**Published:** 2020-12-22

**Authors:** M. E. Stegmann, D. Brandenbarg, A. K. L. Reyners, W. H. van Geffen, T. J. N. Hiltermann, A. J. Berendsen

**Affiliations:** 1 Department of General Practice and Elderly Care Medicine, University Medical Center Groningen, University of Groningen, Antonius Deusinglaan 1, FA 21, 9713 AV Groningen, The Netherlands; 2 Department of Medical Oncology, University Medical Center Groningen, University of Groningen, Groningen, The Netherlands; 3 Department of Pulmonary Diseases, Medical Centre Leeuwarden, Leeuwarden, The Netherlands; 4 Department of Pulmonary Diseases and Tuberculosis, University Medical Center Groningen, University of Groningen, Groningen, The Netherlands

**Keywords:** Goals, Aged, Neoplasms, Decision-making, Palliative care, Primary health care

## Abstract

**Purpose:**

To investigate the treatment goals of older patients with non-curable cancer, whether those goals changed over time, and if so, what triggered those changes.

**Methods:**

We performed a descriptive and qualitative analysis using the Outcome Prioritization Tool (OPT) to assess patient goals across four conversations with general practitioners (GPs) over 6 months. Text entries from electronic patient records (hospital and general practice) were then analyzed qualitatively for this period.

**Results:**

Of the 29 included patients, 10 (34%) rated extending life and 9 (31%) rated maintaining independence as their most important goals. Patients in the last year before death (late phase) prioritized extending life less often (3 patients; 21%) than those in the early phase (7 patients; 47%). Goals changed for 16 patients during follow-up (12 in the late phase). Qualitative analysis revealed three themes that explained the baseline OPT scores (*prioritizing a specific goal*, *rating a goal as unimportant*, and *treatment choices related to goals*). Another three themes related to changes in OPT scores (*symptoms*, *disease course*, and *life events*) and stability of OPT scores (*stable situation*, *disease-unrelated motivation*, and *stability despite symptoms*).

**Conclusion:**

Patients most often prioritized extending life as the most important goal. However, priorities differed in the late phase of the disease, leading to changed goals. Triggers for change related to both the disease (e.g., symptoms and course) and to other life events. We therefore recommend that goals should be discussed repeatedly, especially near the end of life.

**Trial registration:**

OPTion study: NTR5419

## Introduction

Treatment decisions are often complex for older patients with cancer. The expected positive effects of treatment are unclear in this group because most studies of therapeutic efficacy are performed in younger patients [1, 2]. Older patients also tend to be more frail or to have comorbidities that increase the negative effects of treatment [3]. It is therefore advised that health care providers discuss the benefits and harms of available treatment options comprehensively, making efforts to involve patients in treatment decisions and to respect their preferences whenever possible [4]. This is important because treatment guidelines are generally based on optimal survival, whereas not all older patients will value extending life as the main treatment goal [5]. Indeed, other goals are more important for about half of these patients, such as maintaining independence [5]. The importance of explicitly discussing patients’ treatment goals is exemplified by research showing that health care professionals often make incorrect assumptions about these goals [6]. Furthermore, goals may be dynamic and change as illness progresses, particularly for incurable diseases where treatment priorities often change gradually from extending life to optimizing comfort [7]. In a study of patients with non-curable cancer, most patients said they would like to talk to their health care provider about end-of-life care when their health deteriorated [8]. This suggests that health deterioration may trigger a change in treatment preference. This could also be inferred from findings that patients who were recently hospitalized are more averse to resuscitation and artificial feeding in presented serious illness scenarios [9]. However, we are aware that no literature describing the course of general goals during a progressive disease. In the present study, we therefore aimed to investigate the treatment goals of older patients with non-curable cancer, to determine if those goals changed over time, and if they did change, what triggered that change.

## Methods

This was a descriptive qualitative study of patients included in the intervention group of the OPTion study (NTR5419), which was conducted between November 2015 and January 2019. The protocol and outcomes of the original randomized controlled trial (RCT) have been published elsewhere [10, 11]. The Institutional Review Board of the University Medical Center Groningen assessed the protocol, and we obtained informed consent from all participants.

### The OPTion RCT

The original OPTion RCT was designed to assess how patient self-efficacy was affected by a structured conversation about goals with a general practitioner prior to making a decision about treatment. Older patients (age ≥ 60 years) with non-curable cancer were included in the period between their diagnosis and treatment decision, and they were excluded if they had hematological cancers, a life expectancy of < 3 months, or could not complete the questionnaires. After the treatment decision (baseline), patients completed questionnaires about demographic characteristics and decision self-efficacy (primary outcome), as well as symptoms of fatigue, depression, and anxiety [11].

**Fig. 1:**
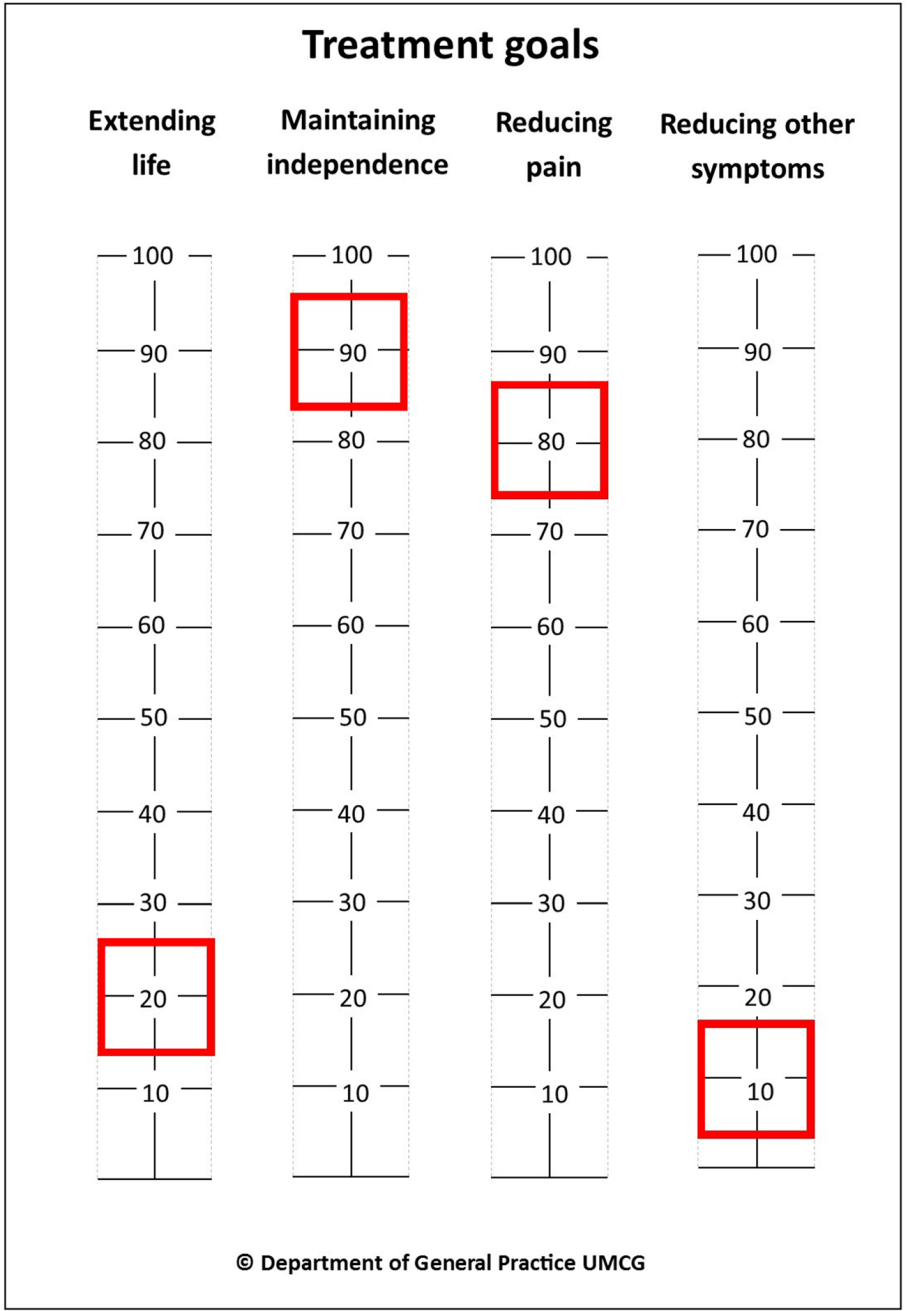
Example of the Outcome Prioritization Tool. Note that in this example of the Outcome Prioritization Tool, the most important goal for this patient was to maintain independence

The Outcome Prioritization Tool (OPT; Fig. 1) was used during the conversation with a GP. This instrument can be used to discuss and prioritize four generic treatment goals: extending life, maintaining independence, reducing pain, and reducing other symptoms [5, 12]. Each goal is valued from 0 to 100, resulting in four OPT scores. Patients were instructed to prioritize the different goals and not rate them as equally important. In the OPTion RCT, patients received either an OPT-facilitated conversation (*n* = 53) or care as usual (*n* = 61). Prioritization discussions in the intervention arm could be facilitated using moveable sliders (0–100). They were provided on a card or were available online (www.optool.nl).

### Current study

In this study, we followed up on the patients included in the intervention group of the OPTion RCT. We repeated the OPT-facilitated GP conversations at 4 weeks, 3 months, and 6 months after baseline and noted the OPT scores. All patients did the follow-up conversations with the same GP as the baseline conversation. Whether former OPT scores were mentioned as a reference was decided by the GP. Text entries made by health care providers were examined in the electronic patient records of hospitals and GP practices from baseline to 6 months. This included both documentation about routine visits and about OPT conversations. In The Netherlands, GPs function as care coordinators and gatekeepers to specialist hospital care. Between hospital visits, most patients also have contact with their GP.

### Data analysis

We descriptively analyzed the OPT scores and their changes over time. In the patient records, we checked if a patient had died and, if so, extracted the date of death. Patients were categorized by their disease phase (early/late) and goal pattern (change/stable). We defined patients as being in the late phase of disease if they died within 1 year of their last follow-up conversation. We defined goals as having changed when the most important treatment goal changed or when at least one value changed by ≥ 20 points (scale 0–100 points) at two consecutive OPT conversations. Thereafter, we analyzed the text entries from patient records in a qualitative hypothesis-generating manner. Two researchers (MES and DB) independently analyzed all text entries using an open coding system, with codes grouped into themes in an axial way. The two researchers discussed and compared their findings until consensus was reached, and a third researcher (AJB) was consulted if disagreement persisted. Representative quotations (*Q*) from the electronic medical records are presented and identified by a patient number in the results. Each quotation is accompanied by the corresponding *OPT scores*, which are displayed as the four scores for extending life, maintaining independence, reducing pain, and reducing other symptoms (i.e., *extending*, *independence*, *pain*, and *others*, respectively).

## Results

### Participants

Of the 47 patients who participated in an OPT conversation at baseline, 29 (62%) had one or more follow-up OPT conversations and could be included in the current study. Among these, the mean age was 75.3 years (standard deviation [SD] = 6.4 years), 24 (83%) were male, 13 (45%) had low education levels, and 17 (61%) had lung cancer (Table 1). About half of the participants (52%; *n* = 15) were in the early phase of the disease. The main reasons for dropout before follow-up were increased illness severity and death. Only 16 patients (55%) attended all three follow-up OPT conversations.

**Table 1:** Patient characteristics, most important goals, and goal patterns

	Total sample (*N* = 29), *N* (%)	Early phase (*N* = 15), *N* (%)	Late phase (*N* = 14), *N* (%)
Age (mean, SD)	75.3 (6.4)	76.4 (6.4)	74.2 (6.3)
Gender: male	24 (83)	12 (80)	12 (86)
Education			
Primary school/GCSE	13 (45)	7 (47)	6 (43)
A-levels	10 (35)	5 (33)	5 (36)
College/University	4 (14)	3 (20)	1 (7)
Not known	1 (10)	0	2 (14)
Cancer type			
Lung	17 (59)	8 (53)	9 (64)
Prostate	4 (14)	1 (7)	3 (21)
Gastrointestinal	6 (21)	4 (27)	2 (14)
Others	2 (7)	2 (13)	0
Hospital			
University hospital	16 (55)	9 (60)	7 (50)
Teaching hospital	5 (17)	2 (13)	3 (21)
Community hospital	8 (28)	4 (27)	4 (29)
Most important goal at baseline			
Extending life	10 (34)	7 (47)	3 (21)
Maintaining independence	9 (31)	4 (27)	5 (36)
Reducing pain	4 (14)	1 (7)	3 (21)
Reducing other symptoms	1 (3)	0	1 (7)
Chose ≥ 1 goal as the most important	5 (17)	3 (20)	2 (14)
Goal pattern			
Changed goals after baseline	16 (55)	4 (27)	12 (86)
Stable goals after baseline	13 (45)	11 (73)	2 (14)

Late phase: death within 1 year of the last follow-up conversation

Changed goal: most important treatment goal changed or ≥ 1 item changed by ≥ 20 points (scale, 0–100) between consecutive OPT-based conversations

### OPT scores

Most patients rated extending life (34%; *n* = 10) or maintaining independence (31%; *n* = 9) as the most important goal at baseline. Patients in the late phase less often rated extending life as the most important goal compared with those in the early phase (3/14 vs 7/15), more often prioritizing the reduction of pain or other symptoms (28% vs 7%) (Table 1). Five patients chose > 1 goal as the most important, despite the OPT guideline explicitly stating that only one should be prioritized. Goals changed during the follow-up period for 16 patients (55%): 4 in the early phase (27%) and 12 in the late phase (86%). For all goals, we observed both increases and decreases in the scores. The most frequently observed change was that extending life became less important in the month before death (21%). However, goals did not change during the follow-up period for 13 patients (45%): 11 in the early phase (73%) and 2 in the late phase (14%).

### Qualitative analysis of electronic patient records

In the qualitative analysis, we reached saturation after reviewing 26 of the 29 records (i.e., no new codes were used for the last three patients) [13]. We deducted three topics: (1) *reasons for a specific baseline OPT score*, (2) *reasons for an OPT score change*, and (3) *reasons for OPT score stability*. For each topic, several themes were identified (Table 2; discussed in the following paragraphs). There were also some differences between GP and specialist records, with the latter having a tendency to report patients’ conditions more positively (quotation1).

Q1a: *Patient says he is feeling pretty good* (Patient number 078, hospital record)

Q1b*: It is not going well. Chemotherapy has much effect on patient* (078, GP record, 1 day later)

OPT scores: 80-80-80-80 (extending-independence-pain-other)

**Table 2:** Topics and themes from the qualitative analysis

Topic	Themes
Reasons for a specific baseline OPT score	Prioritizing a specific goal Rating a goal as unimportant Treatment choices related to goals
Reasons for goal changes during follow-up	Change related to symptoms Change related to disease course Change related to life events
Reasons for goal stability during follow-up	Stability related to stable situation Stability related to disease-unrelated motivation Stability despite symptoms

Changed goal: most important treatment goal changed or ≥ 1 item changed by ≥ 20 points (scale, 0–100) between OPT-based conversations

### Reasons for a specific baseline OPT score

We analyzed 29 patient records regarding the choice of initial goals and found three themes: *prioritizing a specific goal*, *rating a goal as unimportant*, and *treatment choices related to goals* (Table 2). Some patients *prioritized a specific goal* consciously. In their text entries, health care providers described that some patients with a high score for extending life and/or maintaining independence were feeling healthy and wanted to preserve the feeling (Q2). Furthermore, some patients who prioritized maintaining independence explicitly indicated that quality of life was very important to them. This also applied to patients who, according to their patient records, lived very independently with a small social network. Patients with a high OPT score for reducing pain were often those recorded as experiencing pain in their medical records. *Rating a goal as unimportant* was found for one patient in the early phase and one patient in the late phase, with these explicitly stating that extending life was no longer important, consistent with giving low scores in this domain (Q3).

Six patients had several treatment options described in the patient record, and the considerations for the *treatment choice related to goals* were described explicitly for two of them. In both cases, the patient opted to refrain from active treatment after ample consideration of the risks and benefits, as reflected by low scores for extending life (Q4). Both patients also explicitly discussed their treatment options with their GP. In one of these two cases, as well as in another two cases, the medical specialist and GP discussed treatment options and/or goals by phone contact. For most patients, the goal of the chosen treatment matched the goal prioritized by the patients during the OPT conversation. For two patients, however, the goals and chosen treatment did not match: both scored low on extending life, but they chose a life-extending treatment.

Q2: Patient is down to earth and has a clear opinion during the conversation. She feels vital and would very much like to try treatment in order to live longer. (065, GP record)

OPT scores: 100-90-0-0 (extending-independence-pain-other)

Q3: Patient lives alone, does have domestic help, still cooks for himself, does his own grocery shopping, otherwise he spends a large part of the day sitting in a chair. His hips are painful, sagging leg, has not yet received radiotherapy, does not want to (…). Does not want chemotherapy either. (…) “I am 84, what more do you want?” (266, hospital record)

OPT scores: 60-90-80-30 (extending-independence-pain-other)

Q4: Patient is concerned about the side-effects of treatment and their impact on his daily routine. After joint consultation with the geriatrician and me, patient refrains from treatment. He realizes that if we do not treat the tumor now, he will develop symptoms and will probably die due to tumor progression. (238, hospital record)

OPT scores: 30-50-60-60 (extending-independence-pain-other)

### Reasons for goal changes during follow-up

We analyzed 16 patient records in relation to changing goals and inducted three themes: *symptoms*, *disease course*, or *life events* (Table 2). Changes related to s*ymptoms* were observed most often. For patients who lowered their score on maintaining independence, a deterioration in their overall condition was described. According to the text entries, patients who experienced more pain over the course of their treatment changed their goals to give more importance to reducing pain during subsequent follow-up (Q5). Similar explanations were observed for changes in the goal of reducing other symptoms. When patients started to experience fatigue, or when this was aggravated, their goal to reduce other symptoms changed accordingly. One patient changed several goals after having a conversation with his GP, concluding that chemotherapy had several unwanted negative side effects. For changes related to the *disease course*, we observed a decrease in the value of extending life after patients had heard that their disease had progressed despite treatment (Q6). On the contrary, two patients gave a higher value to extending life after they had started a new treatment. Changes related to *life events* were observed for three patients. One was in response to celebrating the birthday of his 102-year-old mother, which resulted in him also wanting to live longer, as reflected by a higher value on this domain (Q7). However, two other patients reported that the death of a close relative lowered their desire to extend life.

Q5: Conversation about the course of events. Patient complains about more fatigue and more pain. (180, GP record)

OPT scores: 30-70-50-50 from 30-80-30-60 (extending-independence-pain-other)

Q6: The CT scan shows progression of the primary tumor (mediastinal, hilar) and progression of the bone metastases. Patient opts for supportive care. The option for another chemotherapy exists, but she has so much pain and has deteriorated so rapidly, that she does not want to continue chemotherapy to possibly slow down the illness, but then probably with so much back pain. (216 hospital record)

OPT scores: 10-60-80-70 from 50-90-80-70 (extending-independence-pain-other)

Q7: Besides short of breath on exertion, he feels very well. His mother just turned 102, he wants that too. (012 GP record)

OPT scores: 80-80-20-20 from 30-30-80-80 (extending-independence-pain-other)

### Reasons for goal stability during follow-up

We analyzed 29 patient records (those who had no changes at all and those who had stable moments) regarding the stability of goals. Three themes related to stability: *stable situation*, *disease-unrelated motivation*, and *stability despite symptom increases* (Table 2). For all patients in a *stable situation*, text entries described that they did not experience changes in disease symptoms, that their condition was generally stable, and that they had few or no treatment-related side effects. As such, there seemed no reason to adjust their goals (Q8). Some (*n* = 2) patients appeared to determine their goals based on some *disease-unrelated motivation* that did not change during follow-up*.* For example, a patient who valued extending life highly said that this was important because of his religion (Q9). In seven patients, we observed *stability despite symptom increases*. According to their medical records, these patients had an increase in disease-related symptoms and/or experienced substantial side effects from therapy. However, they did not adjust their goals or scores related to reducing pain or other symptoms, even when their treatment was changed accordingly (Q10). No reasons were identified.

Q8: No new complaints or insights. Wants to leave the OPT scores as they are. (036, GP record)

OPT scores : 40-60-20-10 (extending-independence-pain-other)

Q9: She [spouse] says that her husband will die when it’s his time. She wants to keep him with her for as long as possible. They will not just “throw in the towel.” (203 hospital record)

OPT scores (extending-independence-pain-other): 90-10-70-40

Q10: Severe back pain (…) Stop chemotherapy, start symptom-oriented palliation. (265 hospital record)

OPT scores: 80-70-30-50 (extending-independence-pain-other)

## Discussion

### Summary

Extending life was most often (*n* = 10, 34%) prioritized as the most important goal. However, compared with patients in the early phase of their disease, those in the late phase not only prioritized extending life less often (*n* = 3, 21% vs *n* = 7, 47%) but also changed their goals more often (*n* = 12, 86% vs *n* = 4, 27%). Triggers for these changes appeared related to both the disease (e.g., symptoms, disease course) and to other life events (e.g., death of a close relative). Goal stability was most often observed in patients without changes in disease symptoms or without side effects from treatment.

### Strengths and limitations

As far as we know, this is the first study exploring the changes over time in the generic treatment goals of patients with non-curable cancer. The qualitative design meant that we could generate hypotheses about which patients change their goals and what might trigger those changes, although these hypotheses must now be validated in further research. An important limitation of the study is that we did not use a purposive sample, which is recommended to achieve a wide variety of participants in qualitative research [14]. Instead, we used the intervention group of the OPTion RCT in which more than half of the participants were men with lung cancer treated in an academic hospital. However, older patients with lung cancer are often less well educated and are generally more difficult to recruit, making this an interesting sample.

Another limitation is that we used text entries from the patient records of health care providers. This is important because these reflect the health care provider’s perception of the consultation, and there will have been incomplete details of what was discussed. For example, although we found few entries concerning the benefits and harms of different treatment options, these may have been discussed but not recorded. Similarly, we could not discuss prior treatment experiences because we lacked that information.

### Comparison with existing literature

Most patients in this study indicated that extending life (34%) or maintaining independence (31%) was their most important goal. Although other studies among older patients have shown similar percentages for extending life (31–35%), they have also shown that maintaining independence was more often prioritized as the most important outcome (35–49%) [5, 15]. This might be explained by patients in our sample having non-curable cancer, in whom there was a notable difference between the early phase (47% and 27% for extending life and maintaining independence, respectively) and the late phase (21% and 36% for extending life and maintaining independence, respectively). The relatively high proportion of patients who prioritized extending life in our early phase group could be explained not only by the longer life expectancy of this group but also the high proportion of patients receiving care in a university hospital (60%). Patients can be referred to this center for new (targeted) therapies in a research context, which may have led to a more select population with a strong wish for treatment.

Our study suggests that patients’ goals become less stable when they enter the late phase of their disease. In practice, it is a challenge for health care providers to determine when patients have entered this phase. The surprise question (“Would I be surprised if this patient died in the next year?”) has been shown to be a simple and effective tool for identifying patients with cancer who have a greatly increased 1-year mortality risk [16]. Recently, it was suggested that a double surprise question, adding “Would it surprise me if this patient is still alive after 12 months?” had even better predictive values [17]. Our research also showed that non-disease-related life events may be a trigger for change, though it remains a challenge to identify such patients who are about to change their goals and may want to adjust their treatment accordingly. This is in line with the widely supported suggestion that health care professionals in the field of palliative care should discuss not only physical issues but also psychological, social, and existential issues [18].

Finally, the GP was asked to perform the OPT conversations in the current study. This was a deliberate choice based on the often longstanding relationship between GPs and older patients in The Netherlands [11, 19, 20]. In a recent survey, more than two thirds of responding cancer patients indicated a need to discuss their treatment decision with the GP [21]. During this conversation, the GP must explain information, check understanding, and discuss priorities [21]. However, it is important that this supplements, rather than replaces, the discussions that patients have about goals and preferences with medical specialists and/or specialist nurses [5].

## Conclusion

We showed that the treatment goals of older patients with non-curable cancer can change over time and that these changes may be related to the primary disease or to other life events. Future research may benefit from analyzing the audio recordings of consultations or from interviewing patients about their goals and the reasons for changes. In the meantime, however, health care providers should endeavor to discuss goals regularly with their patients. This enables the health care provider to more directly assist the patient in making choices, by linking the discussed goals to the different treatment options. This should certainly occur when there is a change in the disease, but we contend that it may be more prudent to perform this as part of a regular and more comprehensive multidomain assessment. Correctly and regularly identifying patient goals, as well as communicating them with other health care providers, can help ensure that therapy remains appropriate and evolves with the needs of patients.
